# Cortical Structure in Nodes of the Default Mode Network Estimates General Intelligence

**DOI:** 10.1002/brb3.70531

**Published:** 2025-05-12

**Authors:** Abhinav Yadav, Archana Purushotham

**Affiliations:** ^1^ Institute for Stem Cell Science and Regenerative Medicine Bangalore India; ^2^ National Centre for Biological Sciences Bangalore India; ^3^ University of Trans‐Disciplinary Health Sciences and Technology Bangalore India; ^4^ Michael E. DeBakey VA Houston USA; ^5^ Baylor College of Medicine Houston USA

**Keywords:** cortical thickness, default mode network, general intelligence, local gyrification index

## Abstract

**Introduction:**

A growing number of studies implicate functional brain networks in intelligence, but it is unclear if network nodal structure relates to intelligence.

**Methods:**

Using MRI, we studied the relationship of the general intelligence factor (g) with cortical thickness (CT), local gyrification index (LGI), and voxel‐based morphometry in the nodes of the default mode network (DMN) and task‐positive network (TPN) in a cohort of 44 young, healthy adults. Employing a novel strategy, we performed repeated analyses with multiple sets of g estimates to remove false positives.

**Results:**

CT and LGI in medial and temporal nodes of the DMN were reliably correlated with g (p < 0.05; Pearson's coefficient: ‑0.52 to ‑0.25 and 0.22 to 0.41, respectively). Linear regression models were developed with these parameters to estimate individual g scores, with a median adj. R^2^ of 0.25.

**Conclusion:**

Cortical thickness and gyrification in key nodes of the Default Mode Network correlate with intelligence. Linear regression models with these cortical parameters may provide an estimate of the g factor.

## Graphical abstract

In young, healthy adults, we found that cortical morphology ‐ specifically, the cortical thickness and local gyrification index ‐ in key medial and temporal nodes of the default mode network, correlated reliably with the general intelligence (g) factor. These cortical parameters may be used in a regression model to estimate the g‐factor.

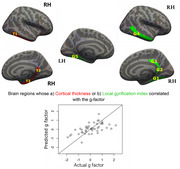

## 1 Introduction

The study of intelligence has generated much interest, but its neural basis is yet to be fully elucidated. With the discovery of intrinsic or resting‐state networks in the brain, cerebral functioning has come to be increasingly viewed in terms of networks. The default mode network (DMN) is a functional brain network that exhibits activity during the resting state (Raichle et al. 2001, Raichle 2015), while the task positive network (TPN) exhibits increased activity in response to cognitive tasks, particularly those requiring attention (Corbetta and Shulman 2002, Fox et al. 2005). There is growing evidence that the properties of these networks are correlated with intelligence.

The global efficiency of the DMN (Song et al. 2009) and nodal efficiency in the TPN (Hilger et al. 2017) have been found to correlate with intelligence. Segregation between the DMN and other resting‐state networks is associated with higher intelligence both in older adults and in children (Lindbergh et al. 2019, Sherman et al. 2014). The strength, stability, and variability of connectivity spatial patterns in multiple networks, including the DMN and dorsal attention network among others, predict cognitive performance (Liu et al. 2018).

Data suggests that intelligence may be reflected in the nodal structural characteristics of these networks as well. In mathematically gifted adolescents, cortical thickness (CT) is decreased in the superior frontal gyrus, precuneus, and medial pre‐frontal cortex compared to age‐ and IQ‐matched controls. Similarly, the cortical surface area (SA) is increased in the superior frontal and lingual gyri, and the inferior parietal lobule, among other regions (Navas‐Sánchez et al. 2016). Cortical surface complexity in the superior temporal gyrus has been reported to positively correlate with numerical intelligence in young adults (Heidekum et al. 2020). In multivariate analyses, positive canonical cross‐loadings with intelligence represented by the g‐factor have been reported for cortical SA in the superior temporal, insula, middle frontal, and fusiform gyri in adolescents (Modabbernia et al. 2021). In a mixed cohort of children, adolescents, and adults, IQ correlated with CT in the orbitofrontal cortices, supramarginal gyri, superior temporal gyri, and left lingual and parahippocampal gyri (Schmitt et al. 2019). Early adolescents with greater CT in the precuneus, posterior cingulate, insula, superior frontal, superior and inferior temporal gyri among others, went on to become high academic achievers in high school (Meruelo et al. 2019). In light of these findings, we hypothesized that cortical structure in the nodes of the DMN and TPN would reflect differences in intelligence in healthy adults.

The general intelligence (g) factor—a construct put forth by Spearman in 1904 ‐ has been defined as the common intelligence that underlies cognitive abilities across multiple domains (Spearman 1904, McGrew 1997, McGrew 2009). The Cattell‐Horn‐Carroll model (Floyd et al. 2009, Karama et al. 2011) of general intelligence postulates three strata of scores, each explaining the variance of the scores from its lower stratum. At the highest level is the “g”, followed by scores specific to each cognitive domain at the middle level, with individual test scores at the basal level. The g factor is therefore calculated by factor analysis of a set of test scores spanning multiple cognitive domains (Spearman 1904, McGrew 1997, McGrew 2009). The quality of g depends on the diversity of the test scores used, but by definition, is robust to the specific combination of tests used (Spearman 1904, Floyd et al. 2009, Major et al. 2011)—a property known as the positive manifold of g. The neurophysiological substrates of g are believed to include nerve conduction velocity, genes, and brain size (Deary et al. 2010). IQ, on the other hand, is a summation of standardized scores, and may be understood as a combination of g plus specific cognitive abilities and skills (Colom et al. 2002).

Lack of reproducibility is a challenge with much of experimental science, including neuroimaging (Ioannidis 2018, Eklund et al. 2016, Poldrack et al. 2017). False‐positive results may contribute to the lack of clear consensus among imaging studies of intelligence. We chose g as our measure of intelligence over other measures such as IQ, as we wanted to take advantage of the positive manifold property of g in a novel analysis strategy to exclude false‐positive results. We reasoned that a true neural correlate of the g factor would correlate with g estimates derived from different combinations of test scores due to its positive manifold property. Hence, we derived multiple g estimates using different combinations of test scores from a test battery in a cohort of healthy young adults. We then performed parallel analyses using each set of g estimates, and identified network nodes that showed structural correlation with a majority of these g estimates.

## 2 Methods

### 2.1 Participant Recruitment

This study was approved by the Institutional Ethics Committee of the Institute of Stem Cell Science and Regenerative Medicine and performed in accordance with relevant guidelines and regulations. All subjects were enrolled only after obtaining informed consent with an explanation of the nature of the study.

Healthy individuals between the ages of 22 and 35 years were recruited for cognitive assessment. Participants had to be fluent in English (the language of the test battery). As our test battery was developed and tested on advanced undergraduate and graduate students, we required participants to have at least three years of a college education. Intelligence is known to correlate with years of education, although reverse causality does not appear to be a major factor (Haier et al. 2004). The relatively homogenous education level of our subjects mitigated any influence of education on g. Those with any history of psychiatric or neurological disorders were excluded.

A subset of willing participants aged between 25 and 35 years of age, without contraindications to MRI, underwent scanning. Their handedness was assigned a rank based on the Edinburgh Handedness Inventory (http://www.brainmapping.org/shared/Edinburgh.php).

### 2.2 Behavioral Assessment

A battery consisting of tests of cognitive and behavioral function spanning multiple cognitive domains, was administered over two separate sessions to participants. Domains traditionally used for g factor or IQ estimation i.e., math/logic, language, executive function, and fluency, encompassing nine tests (Table  1), were used to estimate the g factor for this study. We ensured by questioning, that participants were not sleep‐deprived or fatigued prior to being tested.

**TABLE 1:** Tests used to assess subjects’ cognitive abilities.

Domain	Function	Test	Brief description
Executive	Attention switching, response inhibition	Modified trail making test	Modified version of the trail making test (Reitan 1958) using colored circles with numbers. The task was to alternate between two colors while selecting numbers sequentially; numbered circles of a third color served as a distractor.
	Attention, mental speed	Symbol digit modalities test	Substitution for a set of symbols with the digits 1 to 9, using a conversion chart (Smith 1973).
	Response inhibition	Stroop test	Three‐colored English version of the classic Stroop test (Jensen and Rohwer 1966).
Language	Vocabulary	Word puzzles	Single word puzzles, anagrams and word analogies.
	Comprehension	Comprehension	Timed passage reading followed by questions to test comprehension.
Verbal fluency	Phonemic fluency	F‐A‐S test	Freelisting as many words as possible starting with ‘F’, ‘A’, or ‘S’ in one minute (Spreen 1977).
	Semantic fluency	Category fluency	Freelisting as many words as possible from the categories—animals and birds—in one minute (Acevedo et al. 2000, Goodglass and Kaplan 1983).
Math‐Logic	Mathematics	Mathematical puzzles	Test of mathematical ability.
	Logic	Logical reasoning	Test of logical deduction.

### 2.3 MRI

MRI scans were acquired using a 20‐channel RF head coil at a SIEMENS SKYRA 3 Tesla MRI scanner located in HCG Hospital, Bangalore. High‐resolution T1‐weighted images (echo time 3.9 ms, repetition time 8.3 ms, no. of slices 192, field of view 240*240, slice thickness 1 mm) were obtained.

### 2.4 Analysis

#### 2.4.1 Cognitive Data—g Factor Estimation

We used the Cattell‐Horn‐Carroll model (Floyd et al. 2009, Karama et al. 2011) to calculate the g factor. The math, logic, symbol digit modalities, Stroop, and trail making test scores were weighted by time to finish. The test scores were then tested for normality using the Shapiro‐Wilks test. Math and logic test scores were found to have a non‐normal distribution and were log‐transformed to approximate a normal distribution.

The g factor was calculated using the statistical software R (https://www.r‐project.org/) by performing factor analysis (without rotation) of a subset of the test scores. For each round of g factor estimation, we selected four test scores, one from each of the four cognitive domains. Using factor analysis on the 24 (3×2×2×2) such unique combinations of tests possible, 24 sets of g score estimates were obtained.

#### 2.4.2 Voxel‐Based Morphometry, Cortical Thickness, and Gyrification

FSL (https://fsl.fmrib.ox.ac.uk) was used to perform voxel‐based morphometry (VBM) (Ashburner and Friston 2000, Ashburner and Friston 2001). Images were pre‐processed by brain extraction, segmentation, linear registration, normalization and smoothing. These images were then correlated with each of the 24 sets of g scores using the general linear model (GLM), regressing out gender and handedness. Significance levels were set for this voxel‐based comparison by permutation testing and cluster‐wise thresholding with family‐wise error rate correction for multiple comparisons (p_corrected_ < 0.05).

CT and LGI were estimated using Freesurfer (https://surfer.nmr.mgh.harvard.edu). Each T1 image was reconstructed using standard pre‐processing (brain co‐registration, segmentation, surface extraction), and surface vertices were generated (Dale et al. 1999, Fischl et al. 1999, Fischl et al. 1999, Fischl 2012). Before the group level analysis, each surface was resampled and smoothed at 10 mm FWHM. A white matter surface was created at the white‐gray matter interface, while two more surfaces were created over the gray matter: an outer surface that tightly enveloped the cortical surface visible from the outside, and the pial surface that followed the actual contours of the pia mater into the depths of sulci and over the gyri. CT at each vertex was measured as the shortest distance from the white matter to the pial surface. LGI quantifies the amount of cortex folded within the sulci as compared with the visible cortex on the outer surface i.e., the ratio between the pial surface and the outer surface (Schaer et al. 2008).

We used a region of interest (ROI)‐based approach to identifying the nodes of the DMN and TPN from the Destrieux Atlas (Destrieux et al. 2010, Thomas Yeo et al. 2011). Each of the 24 sets of g scores derived was then used in GLM analyses, separately with CT and LGI (cluster‐wise corrected for multiple comparisons, (p_corrected_ < 0.05). Gender and handedness were regressed out as nuisance covariates in all the group‐level analyses.

**FIGURE 1:**
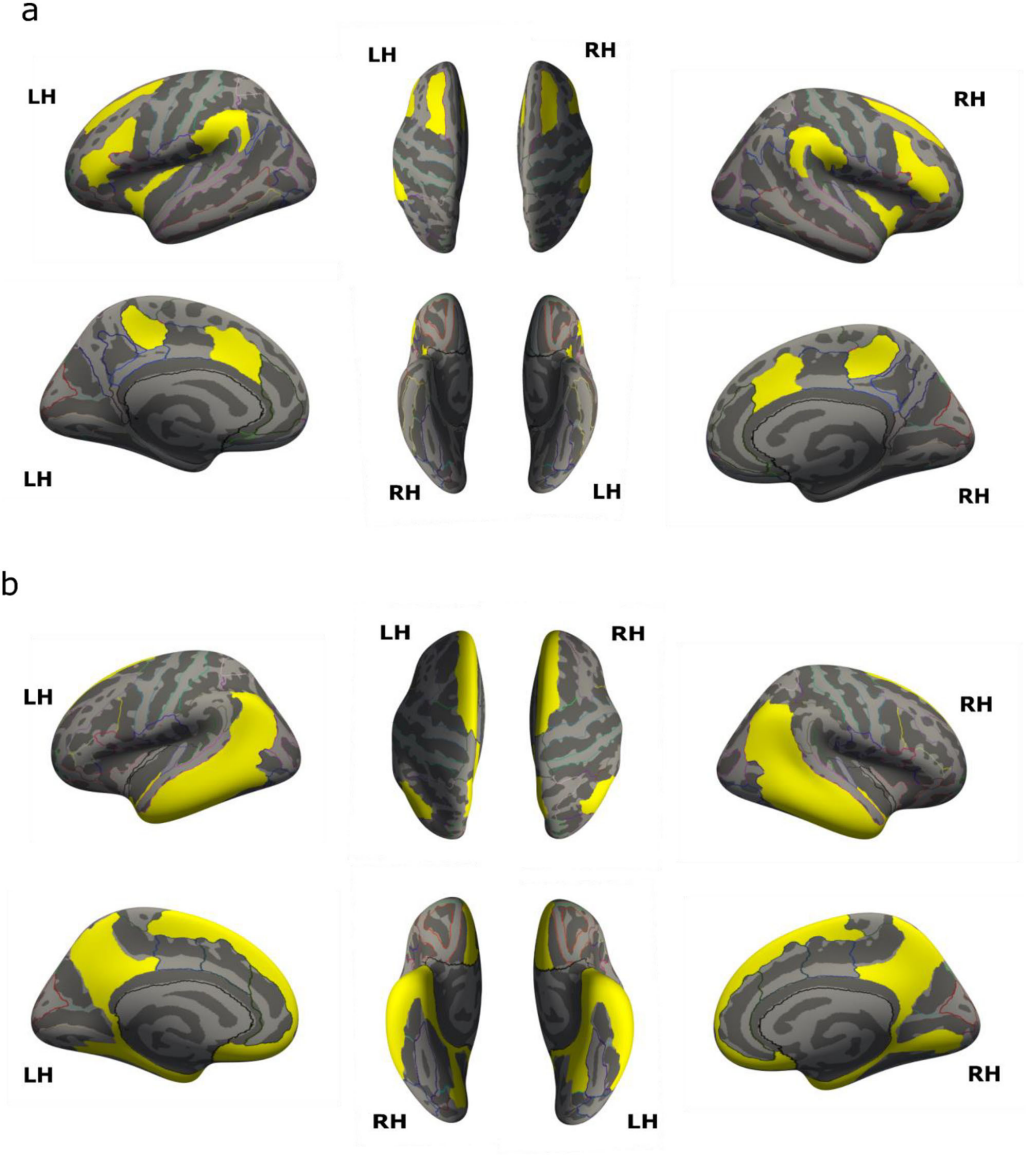
ROIs included in the (a) task positive network and (b) default mode network.

For each ROI, we tabulated which g factors (out of 24) it showed a significant correlation with. We then accepted as significant those ROI's that correlated in > 50%, i.e., at least 13 out of the 24 GLM analyses. ROIs that did not correlate with at least 50% of the g score estimates were rejected as possible false positives. ROI's Included for ‐ TPN : (1) supramarginal gyrus, (2) superior and (3) inferior frontal sulci, (4) triangular inferior frontal gyrus, (5) middle‐anterior cingulate gyrus and sulcus, (6) marginal cingulate sulcus, (7) long insular gyrus and central sulcus of the insula, and (8) short insular gyri (Figure 1a)

DMN : (1) dorsal and (2) ventral posterior cingulate gyri, (3) precuneus, (4) subparietal sulcus, (5) angular gyrus, (6) straight gyrus, (7) subcallosal gyrus, (8) suborbital sulcus, (9) superior frontal gyrus, (10) inferior, (11) middle and (12) planum polare of superior temporal gyri, (13) superior and inferior temporal sulci, (14) temporal pole, (15) collateral sulcus and (16) lingual and medial occipito‐temporal sulcus, and (17) parahippocampal gyrus (Figure 1b)

#### 2.4.3 Regression Model and K‐Fold Validation

The CT and LGI values for ROIs that were significant in the group analysis were then stepwise regressed against the g scores to create a simple linear regression model of the form:

$$Y=\beta_{0}+\beta_{1}X_{1}+\beta_{2}X_{2}+\beta_{3}X_{3}+\cdots +\in$$

where Y is the g score, X_i_ the CT or LGI value, β_i_ the regression coefficient, and ϵ the error term.

We then performed a validation of the model using a K‐fold approach. Matlab (http://www.mathworks.com) was used for the modeling and cross‐validation.

## 3 Results

We enrolled 98 subjects (22–35 years; 45 female; 71 with advanced (post‐graduate) degrees; 88 right‐handed) and assessed them using a cognitive test battery. Scores from nine tests representing four cognitive domains—executive function, math/logic, language, and word generation fluency—were used to derive g scores by factor analysis. 24 sets of g scores were obtained using all possible test combinations covering the four domains. The 24 g scores were mutually highly correlated (Pearson's coefficient: median 0.86; range: 0.71‐0.98), as predicted by the positive manifold phenomenon. (Supplementary Figure)

Of the above cohort, 44 subjects (25–35 years; 16 female; 36 with post‐graduate degrees; 36 right‐handed) also underwent MRI scans. VBM, CT, and LGI maps of the brain were created and used in ROI‐based GLM analyses with each of the 24 sets of g scores. Only those ROIs that correlated with > 12/24 g scores were considered true positives.

### 3.1 Cortical Thickness and G Factor

**FIGURE 2:**
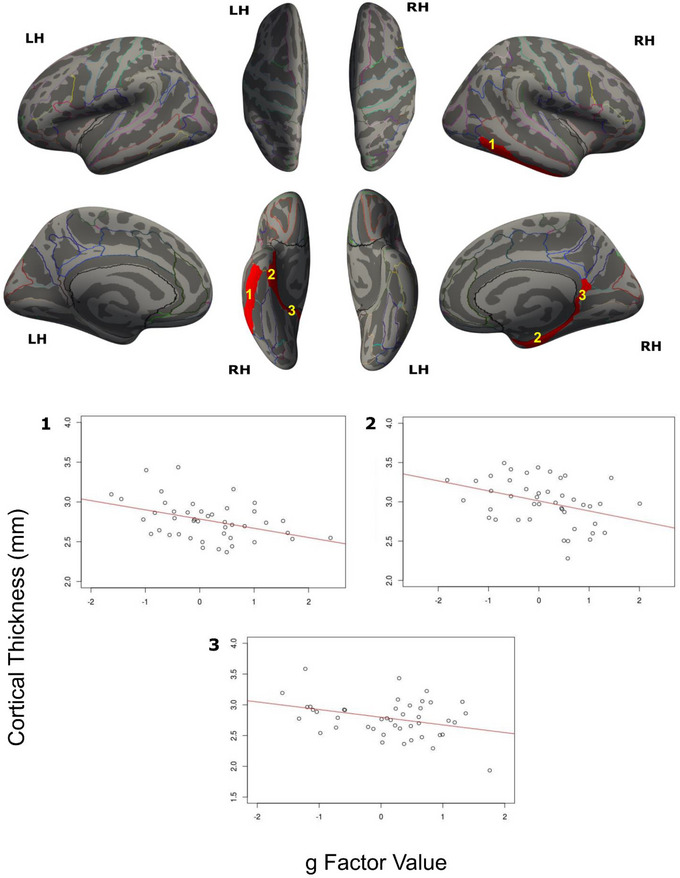
(a) DMN ROIs where CT correlated with g. (1) Right inferior temporal gyrus (cluster‐wise corrected p = 0.0022), (2) right parahippocampal gyrus (p = 0.0050), and (3) right ventral posterior cingulate gyrus (p = 0.0073). (b) Correlation plots of CT versus (median) g scores.

The CT in three nodes of the DMN—inferior temporal gyrus, ventral posterior cingulate gyrus, and parahippocampal gyrus in the right hemisphere significantly correlated with 24, 23, and 20 g scores respectively (Figure 2a), and Pearson's correlation values ranged from ‑0.52 to ‑0.25 (Figure 2b). Clusters in the left inferior temporal gyrus and right collateral and lingual sulci correlated with < 12 g estimates and were rejected.

### 3.2 Local Gyrification Index and G Factor

**FIGURE 3:**
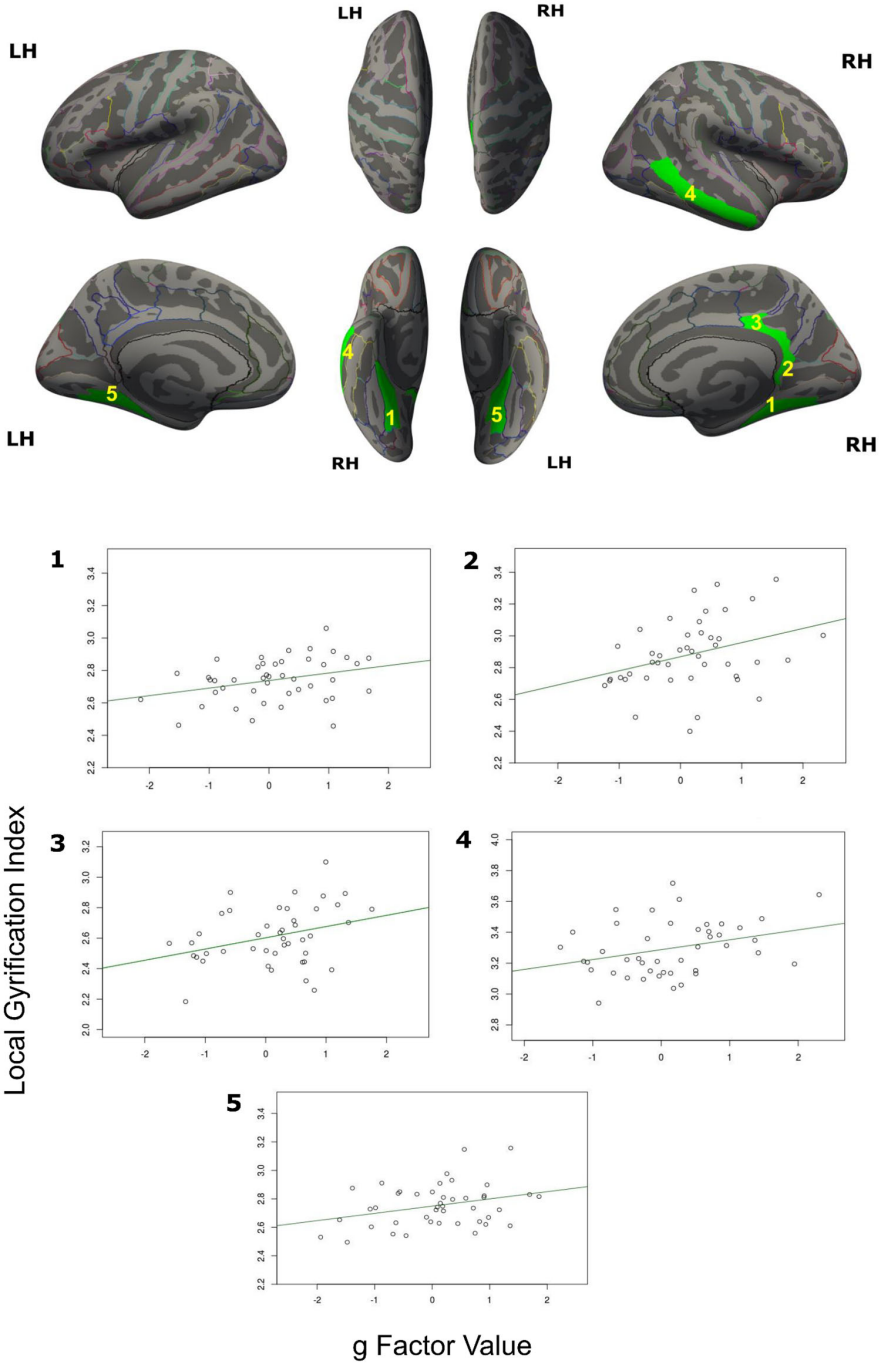
(a) DMN ROIs where LGI correlated with g. (1) Right collateral and lingual sulcus, (2) ventral, (3) dorsal right posterior cingulate gyrus, (4) right middle temporal gyrus, and (5) left collateral and lingual sulcus (cluster‐wise corrected p values: 0.0216, 0.0177, 0.0163, 0.0324, and 0.0172 respectively). (b) Correlation plots of LGI versus (median) g scores.

The LGI in five nodes of the DMN significantly correlated with > 12/24 g estimates by GLM (Figure 3a). The right ventral and dorsal posterior cingulate gyri, right middle temporal gyrus, right and left collateral and lingual sulci correlated with 24, 20, 19, 18, and 16 g scores respectively, with Pearson's correlation values ranging from 0.22 to 0.41 (Figure 3b). The left ventral and dorsal posterior cingulate gyri, right parahippocampal gyrus, right temporal pole, right subparietal, and inferior temporal sulci, and left superior temporal sulcus did not cross the threshold of 12 g‐estimates.

Some nodes of the TPN showed correlations, but with fewer than half the g score estimates. Similarly, the VBM analysis did not identify any nodes that crossed the 12 g score threshold.

We also performed a separate analysis on the 36 right‐handed subjects only, in an attempt to investigate the effect of handedness on the results. In this analysis, the CT in the inferior temporal gyrus, ventral posterior cingulate gyrus and parahippocampal gyrus in the right hemisphere significantly correlated with only 12, 3, and 5 g scores respectively. Similarly, none of the nodes identified in the main analysis crossed the combined significance and “g” score thresholds for LGI, presumably due to the reduced number of subjects. At the same time, no new significant ROI correlations appeared. The small number of left‐handed subjects precluded a separate analysis.

### 3.3 Regression Model and Estimation of G Factor

Stepwise linear regression of each set of g scores with the CT/LGI of the eight brain regions (three regions for CT and five regions for LGI) was performed to create a model for estimation of “g”. The CT of the right inferior temporal gyrus and LGI of right dorsal posterior cingulate gyrus were the most frequently significant parameters across the 24 such regression models of “g”. The regression models could explain a median of 25% of the variance in g scores (adjusted R^2^: 0.15‐0.35).

**FIGURE 4:**
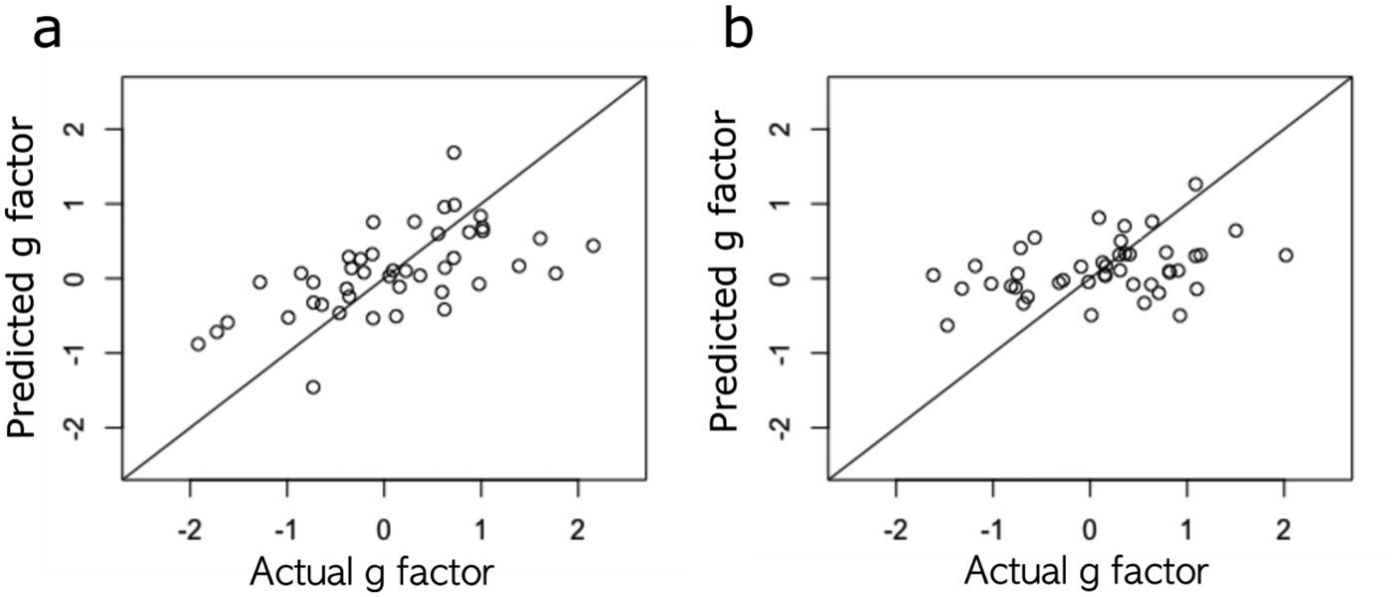
Results of K‐fold analysis to estimate the g factor using a linear model based on CT and LGI of DMN nodes. Predicted versus observed g factor values for the models with the (a) highest and (b) lowest adj. R^2^ values (mean squared error of prediction: 0.411 and 0.485, respectively).

We cross‐validated these regression models using K‐fold analysis, using K = 4. The predicted “g” scores have been plotted against actual scores in Figure 4 for the gfactors with the highest (0.32) and lowest (0.08) adjusted R‐squared values. Excluding outliers, the mean squared errors (MSE) of prediction were 0.411 and 0.485, respectively.

## 4 Discussion

In a cohort of young adults, we show that CT and gyrification index in key nodes of the DMN are reliably correlated with intelligence. Linear regression models using CT and LGI of these nodes of the DMN could explain between 15 and 35% of the variance in g in our sample.

Viewed in light of previous evidence that DMN properties such as global efficiency, segregation from other networks, strength, stability, and variability of connectivity, are related to intelligence (Song et al. 2009, Lindbergh et al. 2019, Liu et al. 2018), our results emphasize the pivotal role of the DMN in intelligence. The variance explained by our DMN‐based model using CT and LGI (15‐35%) is higher than the 11.5% previously reported in adults using LGI alone (Gregory et al. 2016). This also compares favorably with the R^2^ for the well‐known relationship between cerebral volume and intelligence, estimated by meta‐analyses to be of the order of 0.06–0.09 (Pietschnig et al. 2015, Gignac and Bates 2017).

Prior studies—primarily in adolescents—have reported correlations with the nodes of the TPN, especially cortical SA in the inferior parietal lobule (Navas‐Sánchez et al. 2016), CT (Schmitt et al. 2019) in the supramarginal gyrus, CT (Meruelo et al. 2019, Lett et al. 2020) and SA (Modabbernia et al. 2021, Lett et al. 2020) in the insula, and CT and SA in the anterior cingulate (Lett et al. 2020). Another study that included both children and young adults found significant correlations of CT with g in left frontal and parietal areas, right frontal, lateral occipital, and precuneus (Menary et al. 2013).

In contrast, we failed to find any significant structural correlations in the TPN in our adult cohort. Age‐heterogeneous studies in children and adolescents may be more likely to find correlations due to the concomitant effects of age on both cortical morphology and intelligence (Salthouse et al. 2015). For example, both intelligence scores and cortical surface area increase as a child grows, therefore the two would appear to be correlated in a mixed‐age cohort. It is not clear if statistical methods such as regression can completely abrogate these confounding effects.

On the other hand, studies in adults have shown that cortical structure and functional connectivity do not always correlate. In an age‐heterogeneous sample of adults, there was no significant association of functional connectivity with CT (Schulz et al. 2022). In another adult sample, the changes in functional connectivity and CT with age showed regionally varying, positive or negative associations (Vieira et al. 2020). Analysis of gyral and sulcal fMRI signals within different resting‐state networks showed functional heterogeneity of cortical folding patterns between the networks, demonstrating that the relationship between gyrification and functional connectivity is not uniform (Wang et al. 2022). This dissociation between functional and structural properties of resting state networks may explain why we found no nodal structural correlations with intelligence in the TPN. VBM has previously shown no correlation with g, in line with the results we obtained (Haier et al. 2004).

We found a correlation of cortical morphology with g primarily in the temporal and medial nodes of the DMN. A growing body of evidence points to the importance of medial and temporal areas such as the parahippocampus, posterior cingulate, precuneus, fusiform, Broca's and Wernicke's areas in intelligence (Menary et al. 2013, Colom et al. 2009, Karama et al. 2009, Bajaj et al. 2018). The posterior cingulate cortex (PCC) is a central hub of the DMN and shows strong functional connectivity both to the rest of the DMN, and to frontoparietal networks involved in cognitive control (Leech et al. 2012). It is believed to play a key role in directing attention, and in the breadth of attentional focus (Leech and Sharp 2014). The PCC is hypothesized to monitor for changes in the internal and external environments, and to signal a change in behavior in response (Pearson et al. 2011). It is also thought to act as an integrator of information represented in the medial and lateral temporal lobes (Smallwood et al. 2016), and to play an important role in successful memory retrieval (Maddock et al. 2001). Reduced metabolism is seen in this node early on in the course of Alzheimer's disease (Greicius et al. 2004), reaffirming its importance in cognition.

The other nodes we identified are known to have key memory, visual, and language‐related functions. In the resting state, the parahippocampal gyrus is the primary hub of the DMN in the medial temporal lobe. It connects the other nodes of the DMN to the memory system of the medial temporal lobe (Ward et al. 2014). The parahippocampal gyrus also holds a neural representation of topographical space (Aguirre et al. 1996, Epstein and Kanwisher 1998). Functional interactions between the DMN and hippocampal—parahippocampal areas have been found to correlate with the speed of accurate responses (Vatansever et al. 2017).

The inferior temporal cortex is essential for object and pattern recognition, and is a part of the ventral visual pathway (Kravitz et al. 2013, Creem and Proffitt 2001). Inferior temporal lesions can cause visual agnosia (Creem and Proffitt 2001). Recent evidence suggests that inferior temporal neurons are involved in perceptual decision‐making (Dehaqani et al. 2022). The middle temporal gyrus is involved in language functions and in processing semantic memory (Cabeza and Nyberg 2000, Chao et al. 1999), while both the middle and inferior temporal gyri are involved in multi‐sensory integration (Onitsuka et al. 2004).

There are only a few studies of intelligence that have investigated LGI as a cortical structural feature. Correlations with g have been previously reported for LGI in the cingulate cortex and medial temporo‐occipital junction among other areas (Gregory et al. 2016). In line with previous research (Navas‐Sánchez et al. 2016, Tadayon et al. 2020), we found that cortical thinning and greater gyrification correlated with higher intelligence. It has been theorized that learning involves pruning of neuronal connections and creation of a more structured neural network, leading to decreased CT. On the other hand, greater gyrification would mean an increase in the number of cortical columns—the information processing units in the cortex—and allow for increased functional specificity of columns (Tadayon et al. 2020).

Intelligence is known to be heritable, and hundreds of genetic loci have been found to be associated with intelligence (Lett et al. 2020). Cortical morphology may serve as the intermediate structural phenotype that then translates to the functional phenotype of intelligence. Polygenic scores for intelligence derived from a large meta‐analysis of genome‐wide association studies explained 3–5% of the variance in the g factor. This association between polygenic scores and the g‐factor was found to be mediated by cortical morphology, particularly CT and SA in the medial temporal, among other regions (Lett et al. 2020).

We employed the positive manifold property of g to ensure our results were reliable. We derived multiple sets of g estimates and accepted only regions that showed correlation with more than half of these. Had we used a single g estimate as most studies do, we may have reported some of the regions that we rejected, as significant. On the other hand, it is possible our threshold of correlation with at least 50% of g‐score estimates may have been too stringent. However, to ensure a high degree of reliability and confidence in the results obtained, we chose 50% as a reasonable threshold to employ.

We examined which of the 24 sets of g scores correlated with cortical morphology in each node to identify if there was a subset of the scores that tended to show correlations and not others. All 24 sets of g scores showed significant correlations with at least four out of the eight ROIs. Twenty two out of the 24 sets showed correlations with at least five out of 8 ROIs. The correlations we identified thus appear to truly be correlations with general intelligence, and not specific only to some particular combinations of tests.

The relatively small size of our cohort limited our ability to cross‐validate the model we developed. Though we used a K‐fold analysis to investigate the estimability of g using CT and LGI in the DMN, validating this in an independent test cohort would be an important next step. Although our study focused solely on the cortical morphological correlates of general intelligence, the relationship between intelligence and the functional networks of the brain is much more complex. Incorporating both structural and functional data into future studies would provide a more nuanced and comprehensive picture of the network underpinnings of intelligence.

## 5 Conclusion

CT and LGI in key medial and temporal nodes of the DMN are reliably associated with intelligence. Regression models using CT and LGI in these nodes may be used to estimate the general intelligence factor in individual subjects. The DMN appears to play a pivotal role in intelligence.

## Author Contributions

**Abhinav Yadav**: data curation, formal analysis, investigation, methodology, writing – original draft. **Archana Purushotham**: conceptualization, funding acquisition, supervision, writing–original draft, writing – review and editing.

## Ethics Approval Statement

This study was approved by the Institutional Ethics Committee of the Institute of Stem Cell Science and Regenerative Medicine and performed in accordance with relevant guidelines and regulations.

### Peer Review

The peer review history for this article is available at https://publons.com/publon/10.1002/brb3.70531.

## Supporting information



Supporting Information

## Data Availability

Due to privacy concerns, the data of this study will be made available to individual researchers on request, subject to the approval of the relevant Institutional Ethics Committee.

## References

[brb370531-bib-0001] Acevedo, A. , D. A. Loewenstein , W. W. Barker , et al. 2000. “Category Fluency Test: Normative Data for English‐ and Spanish‐Speaking Elderly.” Journal of the International Neuropsychological Society 6: 760–769. 11105466 10.1017/s1355617700677032

[brb370531-bib-0002] Aguirre, G. K. , J. A. Detre , D. C. Alsop , and M. D'Esposito . 1996. “The Parahippocampus Subserves Topographical Learning in Man.” Cerebral Cortex 6: 823–829. 8922339 10.1093/cercor/6.6.823

[brb370531-bib-0003] Ashburner, J. , and K. J. Friston . 2000. “Voxel‐Based Morphometry—The Methods.” Neuroimage 11: 805–821. 10860804 10.1006/nimg.2000.0582

[brb370531-bib-0004] Ashburner, J. , and K. J. Friston . 2001. “Why Voxel‐Based Morphometry Should Be Used.” Neuroimage 14: 1238–1243. 11707080 10.1006/nimg.2001.0961

[brb370531-bib-0005] Bajaj, S. , A. Raikes , R. Smith , et al. 2018. “The Relationship Between General Intelligence and Cortical Structure in Healthy Individuals.” Neuroscience 388: 36–44. 30012372 10.1016/j.neuroscience.2018.07.008

[brb370531-bib-0006] Cabeza, R. , and L. Nyberg . 2000. “Imaging Cognition II: An Empirical Review of 275 PET and fMRI Studies.” Journal of Cognitive Neuroscience 12: 1–47. 10.1162/08989290051137585 10769304

[brb370531-bib-0007] Chao, L. L. , J. V. Haxby , and A. Martin . 1999. “Attribute‐Based Neural Substrates in Temporal Cortex for Perceiving and Knowing About Objects.” Nature Neuroscience 2: 913–919. 10491613 10.1038/13217

[brb370531-bib-0008] Colom, R. , F. J. Abad , L. F. García , and M. Juan‐Espinosa . 2002. “Education, Wechsler's Full Scale IQ, and G.” Intelligence 30: 449–462.

[brb370531-bib-0009] Colom, R. , R. J. Haier , K. Head , et al. 2009. “Gray Matter Correlates of Fluid, Crystallized, and Spatial Intelligence: Testing the P‐FIT Model.” Intelligence 37: 124–135.

[brb370531-bib-0010] Corbetta, M. , and G. L. Shulman . 2002. “Control of Goal‐Directed and Stimulus‐Driven Attention in the Brain.” Nature Reviews Neuroscience 3: 201–215. 11994752 10.1038/nrn755

[brb370531-bib-0011] Creem, S. H. , and D. R. Proffitt . 2001. “Defining the Cortical Visual Systems: ‘What’, ‘Where’, and ‘How’.” Acta Psychology 107: 43–68. 10.1016/s0001-6918(01)00021-x 11388142

[brb370531-bib-0012] Dale, A. M. , B. Fischl , and M. I. Sereno . 1999. “Cortical Surface‐Based Analysis: I. Segmentation and Surface Reconstruction.” Neuroimage 9: 179–194. 9931268 10.1006/nimg.1998.0395

[brb370531-bib-0013] Deary, I. J. , L. Penke , and W. Johnson . 2010. “The Neuroscience of Human Intelligence Differences.” Nature Reviews Neuroscience 11: 201. 20145623 10.1038/nrn2793

[brb370531-bib-0014] Dehaqani, M.‐R. A. , N. Emadi , A.‐H. Vahabie , A. Zandvakili , and H. Esteky . 2022. “Neural Signature of the Perceptual Decision in the Neural Population Responses of the Inferior Temporal Cortex.” Scientific Reports 12: 1–15. 35606516 10.1038/s41598-022-12236-y PMC9127116

[brb370531-bib-0015] Destrieux, C. , B. Fischl , A. Dale , and E. Halgren . 2010. “Automatic Parcellation of Human Cortical Gyri and Sulci Using Standard Anatomical Nomenclature.” Neuroimage 53: 1–15. 20547229 10.1016/j.neuroimage.2010.06.010 PMC2937159

[brb370531-bib-0016] Eklund, A. , T. E. Nichols , and H. Knutsson . 2016. “Cluster Failure: Why fMRI Inferences for Spatial Extent Have Inflated False‐Positive Rates.” PNAS 113: 7900–7905. 27357684 10.1073/pnas.1602413113 PMC4948312

[brb370531-bib-0017] Epstein, R. , and N. Kanwisher . 1998. “A Cortical Representation of the Local Visual Environment.” Nature 392: 598–601. 9560155 10.1038/33402

[brb370531-bib-0018] Fischl, B. 2012. “FreeSurfer.” Neuroimage 62: 774–781. 22248573 10.1016/j.neuroimage.2012.01.021 PMC3685476

[brb370531-bib-0019] Fischl, B. , M. I. Sereno , and A. M. Dale . 1999. “Cortical Surface‐Based Analysis: II: Inflation, Flattening, and a Surface‐Based Coordinate System.” Neuroimage 9: 195–207. 9931269 10.1006/nimg.1998.0396

[brb370531-bib-0020] Fischl, B. , M. I. Sereno , R. B. H. Tootell , and A. M. Dale . 1999. “High‐Resolution Intersubject Averaging and a Coordinate System for the Cortical Surface.” Human Brain Mapping 8: 272–284. 10619420 10.1002/(SICI)1097-0193(1999)8:4<272::AID-HBM10>3.0.CO;2-4 PMC6873338

[brb370531-bib-0021] Floyd, R. G. , E. I. Shands , F. A. Rafael , R. Bergeron , and K. S. McGrew . 2009. “The Dependability of General‐Factor Loadings: The Effects of Factor‐Extraction Methods, Test Battery Composition, Test Battery Size, and Their Interactions.” Intelligence 37: 453–465.

[brb370531-bib-0022] Fox, M. D. , A. Z. Snyder , J. L. Vincent , and M. E. Raichle . 2005. “The Human Brain Is Intrinsically Organized Into Dynamic, Anticorrelated Functional Networks.” PNAS 102: 9673–9678. 15976020 10.1073/pnas.0504136102 PMC1157105

[brb370531-bib-0023] Gignac, G. E. , and T. C. Bates . 2017. “Brain Volume and Intelligence: The Moderating Role of Intelligence Measurement Quality.” Intelligence 64: 18–29.

[brb370531-bib-0024] Goodglass, H. , and E. Kaplan . 1983. “The Assessment of Aphasia and Related Disorders.” In Dictionary of Biological Psychology. Lea & Febiger.

[brb370531-bib-0025] Gregory, M. D. , J. S. Kippenhan , D. Dickinson , et al. 2016. “Regional Variations in Brain Gyrification Are Associated With General Cognitive Ability in Humans.” Current Biology 26: 1301–1305. 27133866 10.1016/j.cub.2016.03.021 PMC4879055

[brb370531-bib-0026] Greicius, M. D. , G. Srivastava , A. L. Reiss , and V. Menon . 2004. “Default‐Mode Network Activity Distinguishes Alzheimer's Disease From Healthy Aging: Evidence From Functional MRI.” PNAS 101: 4637–4642. 15070770 10.1073/pnas.0308627101 PMC384799

[brb370531-bib-0027] Haier, R. J. , R. E. Jung , R. A. Yeo , K. Head , and M. T. Alkire . 2004. “Structural Brain Variation and General Intelligence.” Neuroimage 23: 425–433. 15325390 10.1016/j.neuroimage.2004.04.025

[brb370531-bib-0028] Heidekum, A. E. , S. E. Vogel , and R. H. Grabner . 2020. “Associations Between Individual Differences in Mathematical Competencies and Surface Anatomy of the Adult Brain.” Frontiers in Human Neuroscience 14: 116. 32292335 10.3389/fnhum.2020.00116 PMC7118203

[brb370531-bib-0029] Hilger, K. , M. Ekman , C. J. Fiebach , and U. Basten . 2017. “Efficient Hubs in the Intelligent Brain: Nodal Efficiency of Hub Regions in the Salience Network Is Associated With General Intelligence.” Intelligence 60: 10–25.

[brb370531-bib-0030] Ioannidis, J. P. A. 2018. “Why Most Published Research Findings Are False.” In Getting to Good: Research Integrity in the Biomedical Sciences. PLOS. 10.1371/journal.pmed.0020124.

[brb370531-bib-0031] Jensen, A. R. , and W. D. Rohwer . 1966. “The Stroop Color‐Word Test: A Review.” Acta Psychology 25: 36–93. 10.1016/0001-6918(66)90004-7 5328883

[brb370531-bib-0032] Karama, S. , Y. Ad‐Dab'bagh , R. J. Haier , et al. 2009. “Positive Association Between Cognitive Ability and Cortical Thickness in a Representative US Sample of Healthy 6 to 18 Year‐Olds.” Intelligence 37: 145–155. 20161325 10.1016/j.intell.2008.09.006 PMC2678742

[brb370531-bib-0033] Karama, S. , R. Colom , W. Johnson , et al. 2011. “Cortical Thickness Correlates of Specific Cognitive Performance Accounted for by the General Factor of Intelligence in Healthy Children Aged 6 to 18.” Neuroimage 55: 1443–1453. 21241809 10.1016/j.neuroimage.2011.01.016 PMC3070152

[brb370531-bib-0034] Kravitz, D. J. , K. S. Saleem , C. I. Baker , L. G. Ungerleider , and M. Mishkin . 2013. “The Ventral Visual Pathway: An Expanded Neural Framework for the Processing of Object Quality.” Trends in Cognitive Sciences 17: 26–49. 23265839 10.1016/j.tics.2012.10.011 PMC3532569

[brb370531-bib-0035] Leech, R. , R. Braga , and D. J. Sharp . 2012. “Echoes of the Brain Within the Posterior Cingulate Cortex.” Journal of Neuroscience 32: 215–222. 22219283 10.1523/JNEUROSCI.3689-11.2012 PMC6621313

[brb370531-bib-0036] Leech, R. , and D. J. Sharp . 2014. “The Role of the Posterior Cingulate Cortex in Cognition and Disease.” Brain 137: 12–32. 23869106 10.1093/brain/awt162 PMC3891440

[brb370531-bib-0037] Lett, T. A. , B. O. Vogel , S. Ripke , et al. 2020. “Cortical Surfaces Mediate the Relationship Between Polygenic Scores for Intelligence and General Intelligence.” Cerebral Cortex 30: 2708. 10.1093/cercor/bhz270 PMC7175009 31828294

[brb370531-bib-0038] Lindbergh, C. A. , Y. Zhao , J. Lv , et al. 2019. “Intelligence Moderates the Relationship Between Age and Inter‐Connectivity of Resting State Networks in Older Adults.” Neurobiology of Aging 78: 121–129. 30925300 10.1016/j.neurobiolaging.2019.02.014

[brb370531-bib-0039] Liu, J. , X. Liao , M. Xia , and Y. He . 2018. “Chronnectome Fingerprinting: Identifying Individuals and Predicting Higher Cognitive Functions Using Dynamic Brain Connectivity Patterns.” Human Brain Mapping 39: 902–915. 29143409 10.1002/hbm.23890 PMC6866558

[brb370531-bib-0040] Maddock, R. J. , A. S. Garrett , and M. H. Buonocore . 2001. “Remembering Familiar People: the Posterior Cingulate Cortex and Autobiographical Memory Retrieval.” Neuroscience 104: 667–676. 11440800 10.1016/s0306-4522(01)00108-7

[brb370531-bib-0041] Major, J. T. , W. Johnson , and T. J. Bouchard . 2011. “The Dependability of the General Factor of Intelligence: Why Small, Single‐Factor Models Do Not Adequately Represent G.” Intelligence 39: 418–433.

[brb370531-bib-0042] McGrew, K. S. 1997. “Analysis of the Major Intelligence Batteries According to a Proposed Comprehensive Gf‐Gc Framework.” In Contemporary Intellectual Assessment: Theories, Tests, and Issues, 119–151. The Guilford Press.

[brb370531-bib-0043] McGrew, K. S. 2009. “CHC Theory and the Human Cognitive Abilities Project: Standing on the Shoulders of the Giants of Psychometric Intelligence Research.” Intelligence 37: 1–10.

[brb370531-bib-0044] Menary, K. , P. F. Collins , J. N. Porter , et al. 2013. “Associations Between Cortical Thickness and General Intelligence in Children, Adolescents and Young Adults.” Intelligence 41: 597–606. 24744452 10.1016/j.intell.2013.07.010 PMC3985090

[brb370531-bib-0045] Meruelo, A. D. , J. Jacobus , E. Idy , T. Nguyen‐Louie , G. Brown , and S. F. Tapert . 2019. “Early Adolescent Brain Markers of Late Adolescent Academic Functioning.” Brain Imaging Behavior 13: 945–952. 29911279 10.1007/s11682-018-9912-2 PMC6298856

[brb370531-bib-0046] Modabbernia, A. , A. Reichenberg , A. Ing , et al. 2021. “Linked Patterns of Biological and Environmental Covariation With Brain Structure in Adolescence: A Population‐Based Longitudinal Study.” Molecular Psychiatry 26: 4905–4918. 32444868 10.1038/s41380-020-0757-x PMC7981783

[brb370531-bib-0047] Navas‐Sánchez, F. J. , S. Carmona , Y. Alemán‐Gómez , et al. 2016. “Cortical Morphometry in Frontoparietal and Default Mode Networks in Math‐Gifted Adolescents.” Human Brain Mapping 37: 1893–1902. 26917433 10.1002/hbm.23143 PMC6867317

[brb370531-bib-0048] Onitsuka, T. , M. E. Shenton , D. F. Salisbury , et al. 2004. “Middle and Inferior Temporal Gyrus Gray Matter Volume Abnormalities in Chronic Schizophrenia: An MRI Study.” American Journal of Psychiatry 161: 1603–1611. 15337650 10.1176/appi.ajp.161.9.1603 PMC2793337

[brb370531-bib-0049] Pearson, J. M. , S. R. Heilbronner , D. L. Barack , B. Y. Hayden , and M. L. Platt . 2011. “Posterior Cingulate Cortex: Adapting Behavior to a Changing World.” Trends in Cognitive Sciences 15: 143–151. 21420893 10.1016/j.tics.2011.02.002 PMC3070780

[brb370531-bib-0050] Pietschnig, J. , L. Penke , J. M. Wicherts , M. Zeiler , and M. Voracek . 2015. “Meta‐Analysis of Associations Between Human Brain Volume and Intelligence Differences: How Strong Are They and What Do They Mean?” Neuroscience and Biobehavioral Reviews 57: 411–432. 26449760 10.1016/j.neubiorev.2015.09.017

[brb370531-bib-0051] Poldrack, R. A. , C. I. Baker , J. Durnez , et al. 2017. “Scanning the Horizon: Towards Transparent and Reproducible Neuroimaging Research.” Nature Reviews Neuroscience 18: 115–126. 28053326 10.1038/nrn.2016.167 PMC6910649

[brb370531-bib-0052] Raichle, M. E. 2015. “The Brain's Default Mode Network.” Annual Review of Neuroscience 38: 433–447. 10.1146/annurev-neuro-071013-014030. 25938726

[brb370531-bib-0053] Raichle, M. E. , A. M. Macleod , A. Z. Snyder , and G. L. Shulman . 2001. “A Default Mode of Brain Function.” PNAS 98: 676–682. 11209064 10.1073/pnas.98.2.676 PMC14647

[brb370531-bib-0054] Reitan, R. M. 1958. “Validity of the Trail Making Test as an Indicator of Organic Brain Damage.” Perceptual and Motor Skills 8: 271–276.

[brb370531-bib-0055] Salthouse, T. A. , C. Habeck , Q. Razlighi , D. Barulli , Y. Gazes , and Y. Stern . 2015. “Breadth and Age‐Dependency of Relations Between Cortical Thickness and Cognition.” Neurobiology of Aging 36: 3020–3028. 26356042 10.1016/j.neurobiolaging.2015.08.011 PMC4609615

[brb370531-bib-0056] Schaer, M. , M. B. Cuadra , L. Tamarit , F. Lazeyras , S. Eliez , and J.‐P. Thiran . 2008. “A Surface‐Based Approach to Quantify Local Cortical Gyrification.” IEEE Transactions on Medical Imaging 27: 161–170. 18334438 10.1109/TMI.2007.903576

[brb370531-bib-0057] Schmitt, J. E. , A. Raznahan , L. S. Clasen , et al. 2019. “The Dynamic Associations Between Cortical Thickness and General Intelligence Are Genetically Mediated.” Cerebral Cortex 29: 4743–4752. 30715232 10.1093/cercor/bhz007 PMC6917515

[brb370531-bib-0058] Schulz, M. , C. Mayer , E. Schlemm , et al. 2022. “Association of Age and Structural Brain Changes With Functional Connectivity and Executive Function in a Middle‐Aged to Older Population‐Based Cohort.” Frontiers in Aging Neuroscience 14: 782738. 35283749 10.3389/fnagi.2022.782738 PMC8916110

[brb370531-bib-0059] Sherman, L. E. , J. D. Rudie , J. H. Pfeifer , C. L. Masten , K. McNealy , and M. Dapretto . 2014. “Development of the Default Mode and Central Executive Networks Across Early Adolescence: A Longitudinal Study.” Developmental Cognitive Neuroscience 10: 148–159. 25282602 10.1016/j.dcn.2014.08.002 PMC4854607

[brb370531-bib-0060] Smallwood, J. , T. Karapanagiotidis , F. Ruby , et al. 2016. “Representing Representation: Integration Between the Temporal Lobe and the Posterior Cingulate Influences the Content and Form of Spontaneous Thought.” PLoS ONE , 1–19. 10.1371/journal.pone.0152272. PMC4821638 27045292

[brb370531-bib-0061] Smith, A. 1973. "Symbol Digit Modalities Test." In Los Angeles. Western Psychological Services.

[brb370531-bib-0062] Song, M. , Y. Liu , Y. Zhou , K. Wang , C. Yu , and T. Jiang . 2009. “Default Network and Intelligence Difference.” IEEE Transactions on Autonomous Mental Development 1: 101–109.

[brb370531-bib-0063] Spearman, C. 1904. “‘General Intelligence,’ Objectively Determined and Measured.” American Journal of Psychology 15: 201. 10.1037/0022-3514.86.1.96 14717630

[brb370531-bib-0064] Spreen, O. , and A. L. Benton . 1977. “ Neurosensory Center Comprehensive Examination for Aphasia: Manual of directions. revised edition .” Vicotria, BC, Canada: Neuropsychology Laboratory, University of Victoria.

[brb370531-bib-0065] Tadayon, E. , A. Pascual‐Leone , and E. Santarnecchi . 2020. “Differential Contribution of Cortical Thickness, Surface Area, and Gyrification to Fluid and Crystallized Intelligence.” Cerebral Cortex 30: 215–225. 10.1093/cercor/bhz082. 31329833 PMC7029693

[brb370531-bib-0066] Thomas Yeo, B. T. , F. M. Krienen , J. Sepulcre , et al. 2011. “The Organization of the Human Cerebral Cortex Estimated by Intrinsic Functional Connectivity.” Journal of Neurophysiology 106: 1125. 21653723 10.1152/jn.00338.2011 PMC3174820

[brb370531-bib-0067] Vatansever, D. , D. K. Menon , and E. A. Stamatakis . 2017. “Default Mode Contributions to Automated Information Processing.” PNAS 114: 12821–12826. 29078345 10.1073/pnas.1710521114 PMC5715758

[brb370531-bib-0068] Vieira, B. H. , C. Rondinoni , and C. E. Garrido Salmon . 2020. “Evidence of Regional Associations Between Age‐Related Inter‐Individual Differences in Resting‐State Functional Connectivity and Cortical Thinning Revealed Through a Multi‐Level Analysis.” Neuroimage 211: 116662. 32088317 10.1016/j.neuroimage.2020.116662

[brb370531-bib-0069] Wang, Q. , S. Zhao , Z. He , et al. 2022. “Modeling Functional Difference Between Gyri and Sulci Within Intrinsic Connectivity Networks.” Cerebral Cortex 33: 933–947. 10.1093/cercor/bhac111 35332916

[brb370531-bib-0070] Ward, A. M. , A. P. Schultz , W. Huijbers , K. R. A. Van Dijk , T. Hedden , and R. A. Sperling . 2014. “The Parahippocampal Gyrus Links the Default‐Mode Cortical Network With the Medial Temporal Lobe Memory System.” Human Brain Mapping 35: 1061–1073. 23404748 10.1002/hbm.22234 PMC3773261

