# Supplementary material for: Cortical Structure in Nodes of the Default Mode Network Estimates General Intelligence
Source: Brain Behav. 2025 May 12;15(5):e70531. doi: 10.1002/brb3.70531 (PMC12069858; doi:10.1002/brb3.70531)
Supplement: Supplementary file 1 — Supporting Information [file BRB3-15-e70531-s001.docx]

**Supplementary Figure**


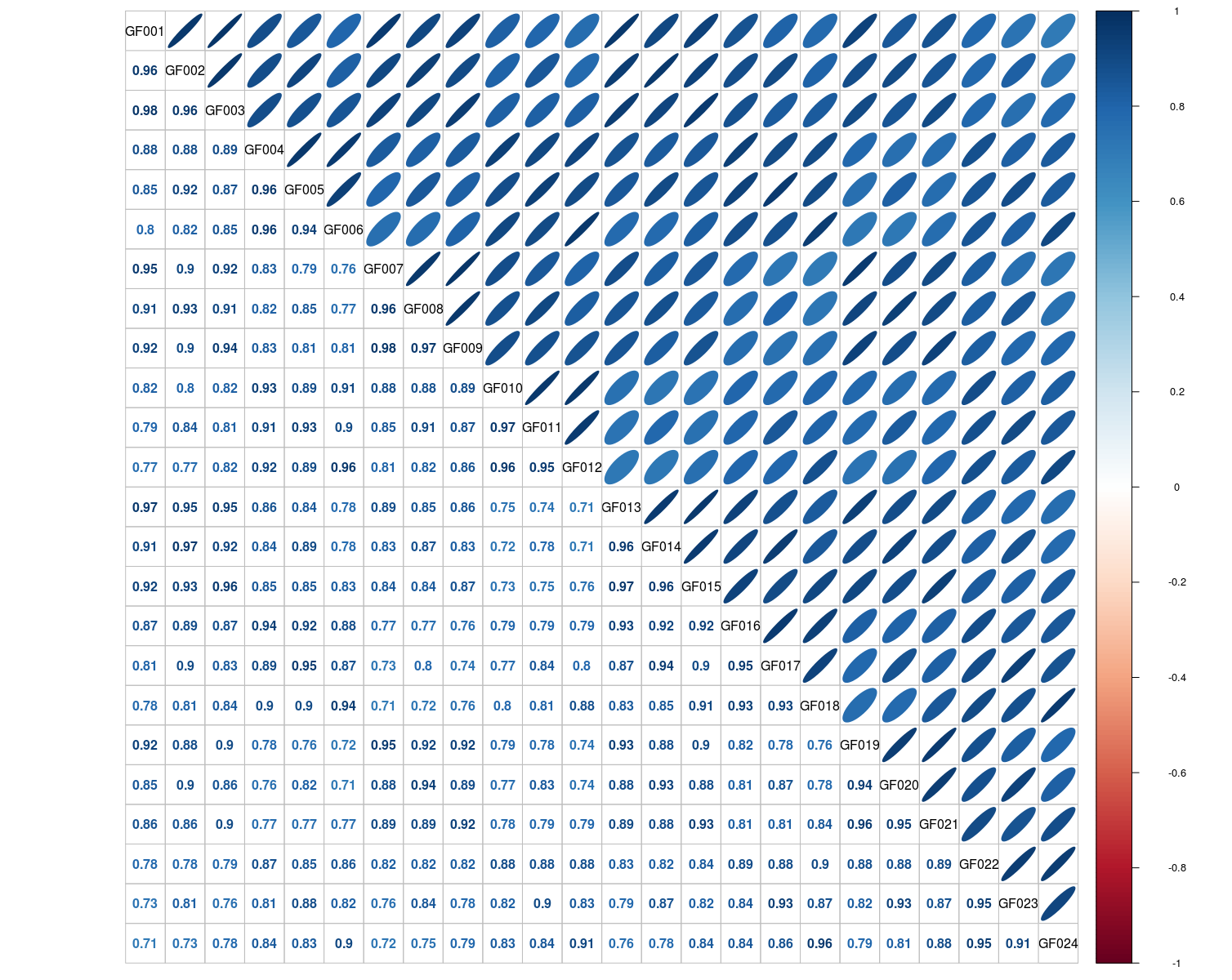


Suppl. Fig: Positive manifold: Pearson’s correlation matrix between 24 sets of g scores
